# Treatment of peri-invagination lesion and vitality preservation in an immature type III dens invaginatus: a case report

**DOI:** 10.1186/s12903-020-1008-x

**Published:** 2020-01-30

**Authors:** Ju-Kyung Lee, Jae Joon Hwang, Hyeon-Cheol Kim

**Affiliations:** 1 Department of Conservative Dentistry, School of Dentistry, Dental Research Institute, Pusan National University, Geumo-ro 20, Mulgeum, Yangsan, Gyeongnam 50612 Korea; 2 Department of Oral and Maxillofacial Radiology, School of Dentistry, Pusan National University, Yangsan, Korea

**Keywords:** Anomaly, Dens Invaginatus, Oehler type III invagination, Peri-invagination periodontitis, Root development, Vitality

## Abstract

**Background:**

To report a case of type III dens invaginatus associated with peri-invagination periodontitis in an immature permanent mandibular central incisor with open apex, in which only the invagination area was treated and vitality was preserved.

**Case presentation:**

A 9-year-old boy was referred complaining of pain in the mandibular left central incisor. After radiographic examination, an invagination into the pulp chamber of the tooth associated with periapical radiolucency was detected. Endodontic access was performed and the orifice was identified under a dental operating microscope. The invagination area was chemo-mechanically cleaned. After 1 week, the invagination was obturated with mineral trioxide aggregate. During the 2-year follow up period, the tooth was asymptomatic. Radiographic examination revealed significant progression of periapical healing and root development in the main root canal of the tooth.

**Conclusion:**

Non-surgical root canal treatment of the invagination may preserve pulp vitality, and continuous root development of the tooth.

## Background

Dens invaginatus (DI) is a developmental anomaly, which results in the deepening or invagination of the enamel organ into the dental papilla before the calcification of dental tissues [1–3]. It is a rare malformation of the teeth, which shows a broad spectrum of morphological variations, in the form and size of the crown and root [3]. Thus, when the pulp complex is infected, cleaning and shaping procedures are more complicated than the usual root canal system [1, 3–5].

While the tooth that is most frequently affected by DI is the permanent maxillary lateral incisor, the occurrence of DI in the mandibular central incisor is rare [5–8]. The generally accepted classification for this anomaly is that proposed by Oehler [9], who categorized it based on the depth of enamel invagination observed radiographically.

A partial invagination limited to the crown of tooth in which the lesion does not extend pass the cementoenamel junction (CEJ) or the pulp was classified to Oehler Type I. In Oehler Type II, the partial invagination extends beyond the crown and CEJ. In type II, pulp may be involved but remain within the root anatomy and there is no communication of the lesion with periodontal ligament. Type III of this classification, in which the invagination continues apically through the root and shows a second foramen into the periodontal tissue, is of particular interest [10]. Oehler Type III invagination is a complete invagination with the lowest incidence amongst the three types [11]. The complex anatomical structures are subdivided into Type III A and Type III B according to the characteristics of communications with the periodontal tissue. Type III A is defined as an invagination extends through the root and communicates laterally with the periodontal ligament space through a pseudo-foramen. There is usually no communication with the pulp, which lies compressed within the root. On the other hand, Type III B invagination extends through the root and communicates with the periodontal ligament at the apical foramen. Neither it has a communication with the pulp [11]. Any infection within the Oehler Type III invagination may lead to an inflammatory response within the periodontal tissues, resulting in peri-invagination periodontitis, irrespective of pulp vitality [10, 12].

Endodontic treatment of the invagination alone is advisable for maintaining the vitality of the main root canal pulp, particularly, if the affected tooth is an immature permanent tooth, with an open apex and vital pulp, while the invagination has a separate apical or lateral foramen. Although there are various management options for these cases, the aim of the treatment should be to maintain pulp vitality of the tooth for further root development.

The present case report presents a case of type III DI associated with peri-invagination periodontitis in an immature permanent mandibular central incisor with open apex, in which only the invagination area was treated. Non-surgical root canal treatment of the invagination preserved vital pulp, and resulted in the resolution of a substantial peri-invagination lesion and continuous root development of the tooth.

## Case presentation

**Fig. 1:**
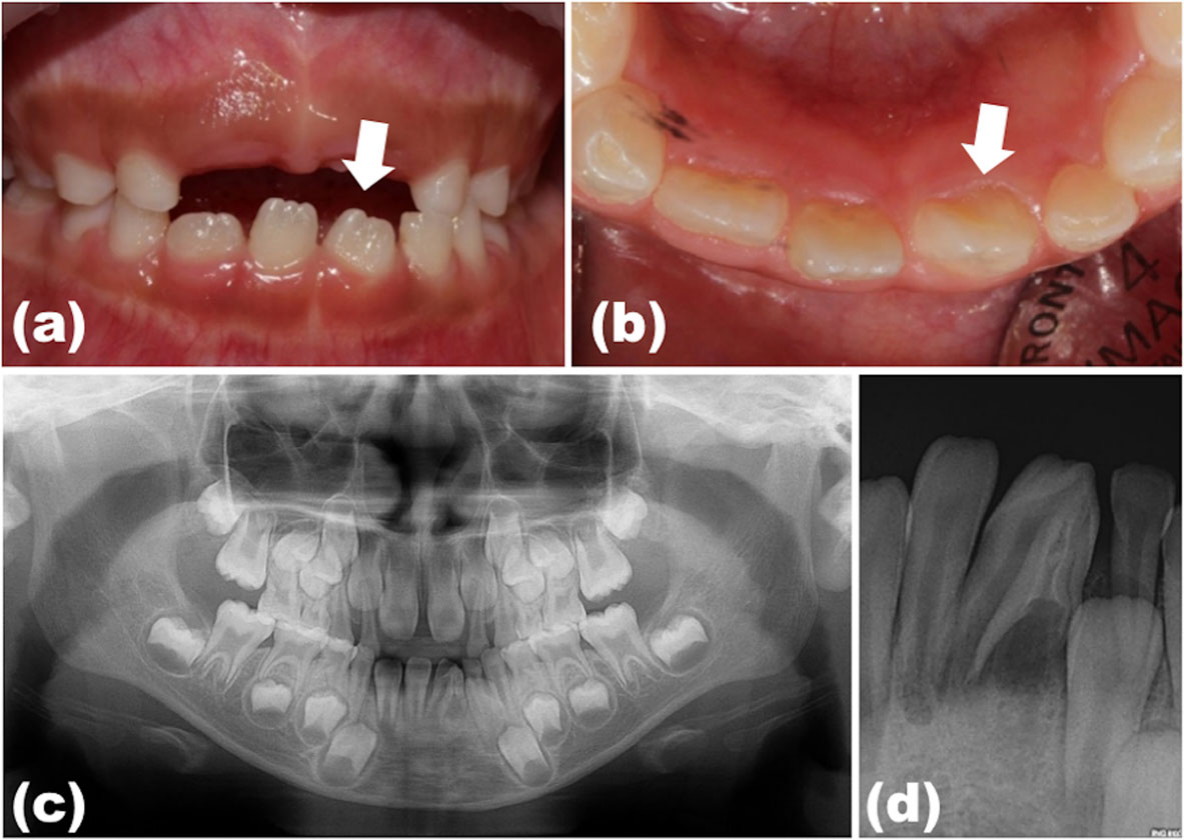
Preoperative photos and radiographs of the dens invaginatus (#31): Clinical photographs show that the mandibular left central incisor (white arrow) was under eruption with coronal anomaly; the clinical crown was slightly larger than the crown of the contralateral tooth (**a** and **b**; labial and incisal views, respectively). (**c**) Panoramic radiograph showing the mandibular dental arch containing the normal number of teeth. (**d**) Periapical radiograph showing an invagination surrounded by a radiopaque enamel border into the pulp chamber associated with periapical radiolucency and incomplete root formation

A 9-year-old boy complaining pain in the mandibular left central incisor was referred from the Department of Pedodontics in Pusan National University Dental Hospital after having clinical examination. He had recently developed acute and spontaneous pain in the region. Clinical examination revealed no intra- and extra-oral swelling. No specific medical, family and psychosocial history were reported. The central incisor was under eruption with a coronal anomaly. The clinical crown had a conical shape and was slightly larger than that of the contralateral tooth (Fig. 1a and b). The periodontal probing depth was within normal range and there was degree-I tooth mobility (< 1 mm horizontal mobility).

**Fig. 2:**
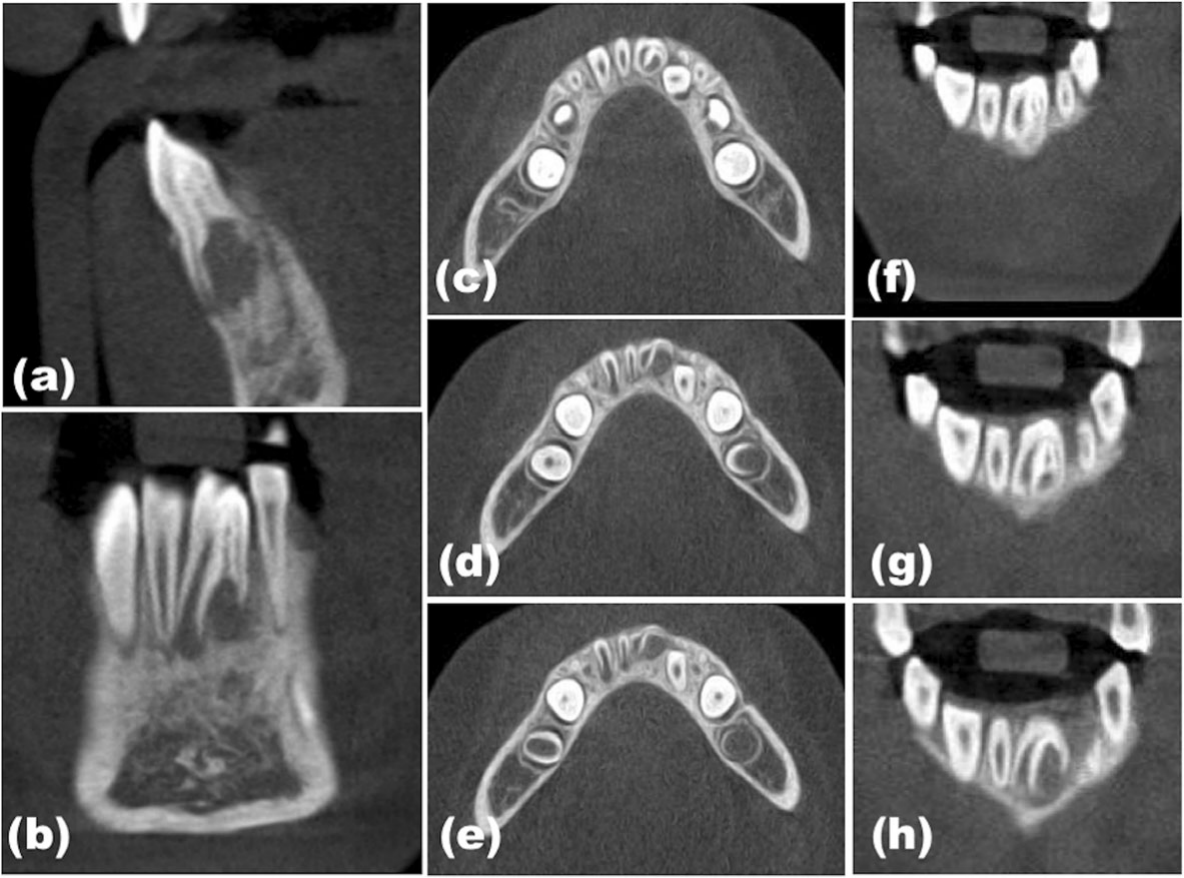
Cone-beam computed tomography images of the dens invaginatus (#31: DI). (**a**) Sagittal and (**b**) coronal views showing the DI with the periapical lesion, surrounded by a radiopaque enamel border in the crown. Axial sections from (**c**, **d**, and **e**) coronal to apical and (**f**, **g** and **h**) coronal sections showing the type III DI extending through the root and communicating through another foramen associated with a periapical radiolucency

Periapical radiography revealed an invagination into the pulp chamber of the tooth and periapical radiolucency with a poorly defined border (Fig. 1c and d). Although the tooth did not respond to an electric pulp vitality test, the reliability of this result was questionable because of its immature root development with open apex. To obtain a more detailed anatomic information and accurate diagnosis, a cone-beam computed tomography (CBCT) (Pax-Zenith 3D; VATECH, Hwaseong, Korea) scan of the involved tooth was performed under 105 KVP, 4.5 mA with 12 cm X 12 cm field of view (Fig. 2).

**Fig. 3:**
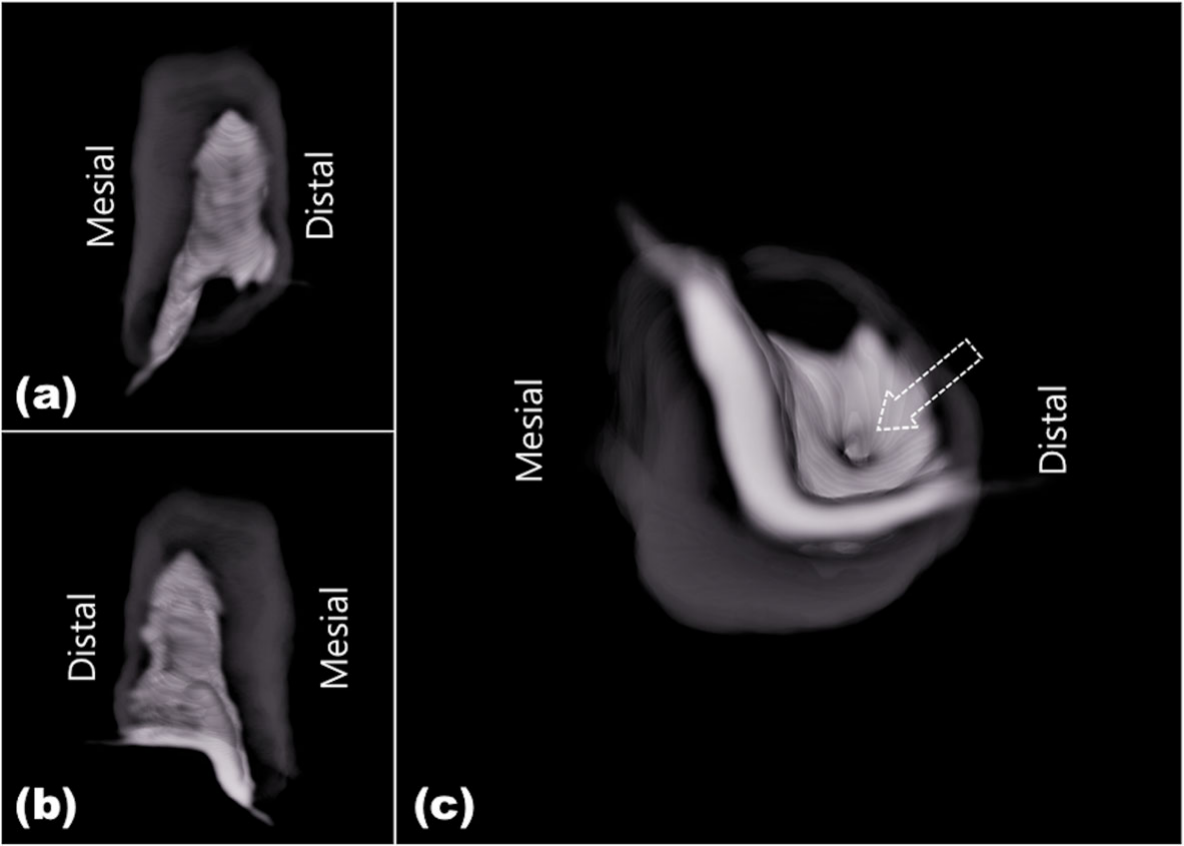
Pre-operative three-dimensional reconstruction aspects of the dens invaginatus (#31: DI) of Oehler type III A. White internal structure is the DI and grey part is the outer part of tooth. Labial view (**a**) and lingual view (**b**) show that the DI is located at distal area. Apical view (**c**) of the DI seen from the apex to crown, shows that DI. Dotted arrow indicates where the invagination communicates with the periodontal tissue. This communication was treated in the resent case

CBCT confirmed the diagnosis of DI with a periapical lesion, surrounded by enamel border in the crown (Fig. 3). The separated invaginated canal was narrow at cervical third and located distal to the main root canal. The invagination extended through the root, communicated through another foramen, had periapical radiolucency, and did not communicate with the main root canal (Figs. 2 and 3). Based on these characteristics, the invagination was classified as Oehler type III A. In the present case, it was postulated that the pain of the tooth might be associated with an infection of the invagination communicated with the periodontal ligament space in isolation to the root canal, which developed the periapical lesion related with the DI, while the pulp remained in vital.

The malformation of the lower incisor made a size discrepancies and the lower dentition was not completely developed on the referral period. Extraction and orthodontic intervention were considered due to the root complexity and unconfident prognosis of conservative treatment. However, the patient’s parent want to keep the natural tooth and pain removal primarily. Patient’s parent agreed to try a conservative treatment with an expectation of potential future treatment of orthodontic and / or prosthetic treatments. The patient’s parent made a signature on the written informed consent form according to the guidelines of the Committee of Institutional Review Board (IRB) and IRB permitted the case report (PNUDH-2019-021).

**Fig. 4:**
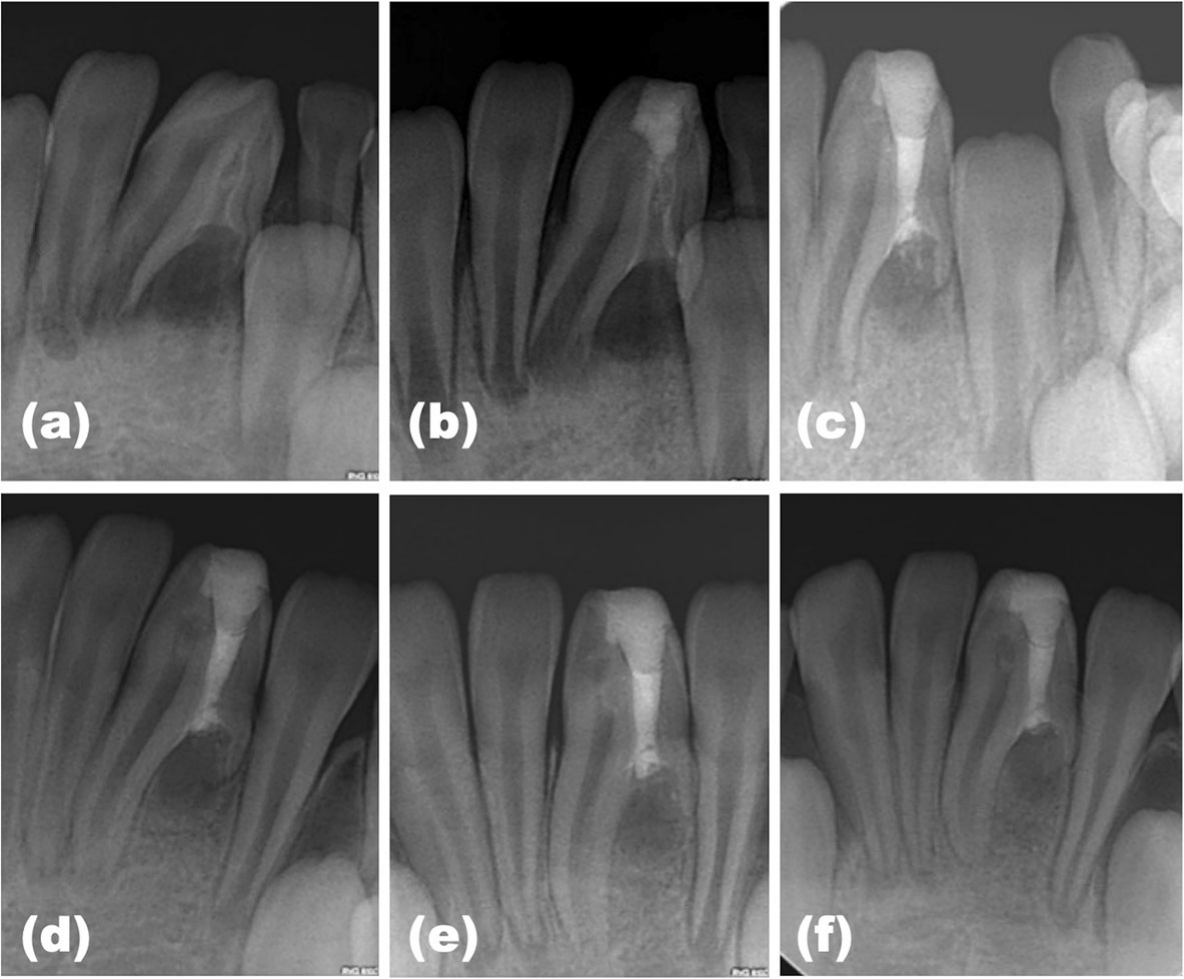
Periapical radiographs during treatment and follow-up periods. (**a**) preoperative, (**b**) after initial treatment with calcium hydroxide paste, (**c**) postoperative after mineral trioxide aggregate filling, and at (**d**) l-year, (**e**) 1.5-year, and (**f**) 2-year recall. There is radiographic evidence of apex closure of the main root canal and root development of the tooth

Under the infiltrative local anaesthesia using one cartridge (1.8 ml) of 2% Lidocaine HCl (1:100,000 epinephrine; Huons, Sungnam, Korea), endodontic access was performed with a small round bur (#2, Komet Dental, Lemgo, Germany) and safe-ended diamond bur (Dia-Burs EX-24; Mani, Tochigi, Japan). After access preparation, rubber dam was applied for tooth isolation. After careful inspection under a dental operating microscope (Leica M320; Leica microsystems, Wetzlar, Germany), the orifice of invagination was identified using an endodontic explorer (DG16 Endo Explorer; Hu-Friedy, Chicago, IL, USA) and #15 K-file (Mani). There was no communication between the main root canal and invaginated canal. Upon access and negotiation of the dens, drainage of purulent and bloody exudation from the periapical tissues was obtained. After the enlargement of the narrow invagination, using a gate-glidden drill (Mani), the working length was determined with radiography and an electronic apex locator (Propex pixi; Dentsply Sirona, Ballaigues, Switzerland). The invaginated lumen was instrumented with stainless-steel hand K-files and nickel-titanium rotary instruments (ProTaper Universal F1, F2 and F3; Dentsply Sirona) with a caution not to over the length. During procedures, the dens was irrigated copiously with 2.5% sodium hypochlorite and saline. Irrigants were agitated using a sonic activated device (Endo-Activator; Dentsply Sirona) to obtain more effective chemical-debridement. The invaginated canal was instrumented to size #60, a calcium hydroxide paste (Calcipex II; Nippon Shika Yakuhin Co., Shimonoseki, Japan) was applied, and the access cavity was sealed with temporary filling material (Caviton; GC Co., Tokyo, Japan). A periapical radiograph was acquired to confirm the canal shape after treatment. The orifice of invagination was very close to the main root canal (Fig. 4b).

At the next appointment after 1 week, it was confirmed that clinical signs and symptoms were disappeared. Therefore, it was determined that the pulp of the main root canal had not been infected and that pulp extirpation and treatment were not required. Following the removal of the intracanal dressing in the invagination, the smear layer was removed through irrigation with ethylenediaminetetraacetic acid (EDTA) solution for 1 min, followed by the application of copious 2.5% sodium hypochlorite solution (NaOCl) using the Endo-Activator. The invaginated canal was then dried with sterilized paper points (Meta Biomed, Cheongju, Korea), before being obturated with mineral trioxide aggregate (MTA; Dentsply Sirona) using a lentulo spiral (Mani) and endodontic pluggers (RCP5/7; Hu-Friedy). A moist cotton plug was placed over the MTA. Access was sealed with a glass ionomer (KetacFil; 3 M ESPE, St. Paul, MN, USA). The patient was recalled after 24 h and the tooth was verified using radiography (Fig. 4c). The access cavity was finally restored with dual-cured composite resin (Luxacore; DMG, Hamburg, Germany).

During the 2-year follow up period, the tooth was asymptomatic and responded normally to pulp vitality testing using ice and an electronic pulp tester. Radiographically, a significant progression of periapical healing was evident, with a substantial reduction in the size of the apical radiolucency (Fig. 4d to f). At the 2-year follow-up, main root development of the tooth was evident with the evidence of apex closure (Fig. 4f).

## Discussion and conclusions

Reports of a non-surgical treatment of the tooth with type III dens invaginatus associated with peri-invagination periodontitis and localized treatment of the invagination are rare in literature. This report presented a case of pulp vitality preservation after the treatment of invagination area to continue the root development of the immature tooth with open apex.

DI is a mal-development of the dental germ, which occurs as a result of the invagination of the enamel organ. If the invagination extends from the crown to the periodontal tissue, but does not communicate with the root canal system, the pulp can remain vital [12]. For Oehlers type III A lesions, especially, it is potentially possible to have peri-invagination periodontitis due to infected invagination [4]. Alani and Bishop [2] recommended that where “peri-invagination periodontitis” exists and the pulp remains healthy all efforts should be aimed at preserving pulp vitality. Tsurumachi [13] also reported a case of type III DI associated with significant peri-invagination periodontitis, in which the invagination alone was treated. In immature permanent teeth, in particular it is very important to maintain pulp vitality because it allows continuous root development, guaranteeing a favourable long-term prognosis with function from matured structure and vitality.

Bacterial contamination of the invagination that occurred subsequent to the eruption of the tooth may result in infection of the invagination, in turn, leading to the development of periapical inflammation and clinical symptoms [12]. In the present case, clinical symptoms and periapical lesion associated with the DI could be managed successfully with exclusive access and treatment of the infected invagination because the invagination was clearly separated from the root canal. During the 2-year follow up period, radiographic evidence of apex closure was obtained. Further, the maintenance of pulp vitality in the main root canal allowed continuous root development of the tooth.

Type III DI is undoubtedly an endodontic challenge due to the complex root canal morphology and the difficulty in accessing irregular and invaginated canals [5]. The absence of an actual apical constriction when the invagination opens into the periodontal tissue contributes to making the successful measuring of working length, cleaning, shaping, and filling challenging [4]. Some areas may be completely untouchable by instrumentation, and thus, proper shaping and cleaning of an irregular volume of the root canal system can be difficult. In the present case, since the invagination had a barrel-shape, limiting the adequate disinfection using mechanical instruments, copious NaOCl irrigation using a sonic activated device was also used. Once root canal preparation is completed, filling procedure using gutta-percha is generally acceptable. However, if the apical anatomy of the root canal is not proper for conventional filling, then MTA would be another optional material. MTA is an appropriate material of choice in the apical area of the invagination because of the invariable funnel shape of the opening [4]. In the present case, the coronal third of the invagination was nearly obliterated and the apical opening had a ‘blunderbuss’ or funnel shape, similar to that found in resorbed or immature apices. The invagination, therefore, was filled with MTA as described at the case presentation. In addition, since the tooth was in process of eruption, there was not enough canal space for backfill, considering that the access cavity should be filled with resin below the expected cementoenamel junction line. Accordingly, the canal was filled entirely with MTA alone.

Advances in contemporary endodontic practice allow clinicians to meet the biological goals of endodontic treatment, in a wide range of clinical situations. The present case report demonstrated the successful diagnosis and conservative treatment of DI, while maintaining pulp vitality of the tooth using contemporary endodontic procedures using a dental operating microscope and CBCT. According to the American Association of Endodontists (AAE) and the American Academy of Oral and Maxillofacial Radiology (AAOMR) Joint Position Statement, the limited fields of view should be considered the CBCT imaging modality of choice for initial treatment of teeth with dental anomalies, especially for the pediatric patients [14]. However, for this case and treatment procedures, the total dentition was scanned to get the information of dental maturation and development as well as the 3-dimensional healing and maturation of the pathosis.

In case of immature permanent tooth with an open apex, in particular, as reported here, the aim of the treatment should be to maintain pulp vitality for further development of the root. If the invagination is necrotic or ‘peri-invagination periodontitis’ is present, but the main canal pulp remains healthy, then the invaginated canal alone has to be treated [12].

Endodontic treatment in teeth of DI, which extends to the apical region and is associated with periapical pathosis, generally involves complicated procedures that require delicate and accurate diagnosis and proper treatment planning. Even in cases of Type III DI combined with a symptomatic periapical lesion, peri-invagination periodontitis can be successfully treated, with the use of contemporary endodontic materials, an operating microscope, and MTA. Maintaining the pulp vitality of the main canal allows root development to continue, guaranteeing long-term prognosis.

## Data Availability

All data generated or analysed which related this case report are included in this published article.

## Abbreviations

AAE: American Association of Endodontists
AAOMR: American Academy of Oral and Maxillofacial Radiology
CBCT: Cone-beam computed tomography
CEJ: Cementoenamel junction
DI: Dens invaginatus
EDTA: Ethylenediaminetetraacetic acid
IRB: Institutional Review Board
MTA: Mineral trioxide aggregate
NaOCl: Sodium hypochlorite solution
